# Data set for fabrication of conformal two-dimensional TiO_2_ by atomic layer deposition using tetrakis (dimethylamino) titanium (TDMAT) and H_2_O precursors

**DOI:** 10.1016/j.dib.2017.06.013

**Published:** 2017-06-09

**Authors:** Serge Zhuiykov, Mohammad Karbalaei Akbari, Zhenyin Hai, Chenyang Xue, Hongyan Xu, Lachlan Hyde

**Affiliations:** a Ghent University Global Campus, Faculty of Bioscience Engineering, Incheon, South Korea; b Key Laboratory of Instrumentation Science and Dynamic Measurement of Ministry of Education, North University of China, 030051, PR China; c School of Materials Science and Engineering, North University of China, 030051, PR China; d Melbourne Centre for Nanofabrication, 151 Wellington Road, Clayton, VIC 3168, Australia

**Keywords:** Atomic layer deposition, Atomic-layered TiO_2_, TDMAT precursor

## Abstract

The data and complementary information presented hare are related to the research article of “http://dx.doi.org/10.1016/j.matdes.2017.02.016; Materials and Design 120 (2017) 99–108” [1]. The article provides data and information on the case of atomic layer deposition (ALD) of ultra-thin two-dimensional TiO_2_ film. The chemical structure of precursors, and the fabrication process were illustrated. The data of spectral ellipsometric measurements and the methods of calculations were presented. Data of root mean square roughness and the average roughness of the ADL TiO_2_ film are presented. The method of bandgap measurements and the bandgap calculation are also explained in the present data article.

**Specifications Table**

**Table:** 

Subject area	*Physics, Materials science*
More specific subject area	*Ultra-thin atomic-layered materials*
Type of data	*Table, image, text file, graph, figure*
How data was acquired	*Spectroscopic ellipsometry, Atomic force Microscopy (AFM)*
Data format	*Analyzed*
Experimental factors	*Development and optimization of fabrication method*
Experimental features	*Thickness measurements, materials characterization, Conductivity*
Data source location	*South Korea, Australia, China, Belgium.*
Data accessibility	*The data are available with this article.*

**Value of Data**

• The information of ALD instrument and samples preparation method can be used by other researchers.
• The data of characterization, optical ellipsometry, AFM measurements and evaluation of optical properties can be used and compared by other methods of thin-film fabrication.
• The data and general approach of experiments can be used as an outlook to lead other researchers for more investigation in the area of atomic-layered films.

## 1 Data

The chemical structure of TDMAT precursors, atomic layer deposition set-up and the final fabricated samples are presented in this data article. The spectral ellipsometry method and measurement techniques are introduced and the final results are presented. Furthermore, the results of atomic force microscopy measurements and the bandgap calculation can be found in present data in brief article.

## 2 Experimental design, materials and methods

### 2.1 Chemical structure of precursor

**Fig. 1:**
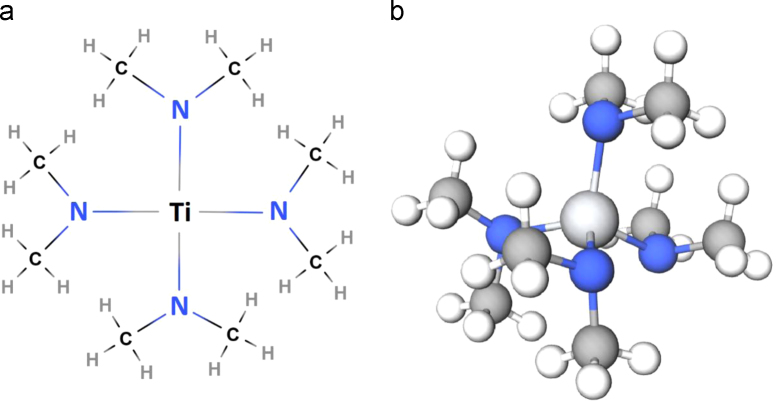
(a) Chemical structure and (b) molecular formula of TDMAT.

Tetrakis (dimethylamino) titanium (TDMAT) is one of the most common used precursors for atomic layer deposition of Titanium-based films. The chemical structure and molecular formula are shown in Fig. 1.

### 2.2 The fabricated sample, atomic layer deposition set-up

**Fig. 2:**
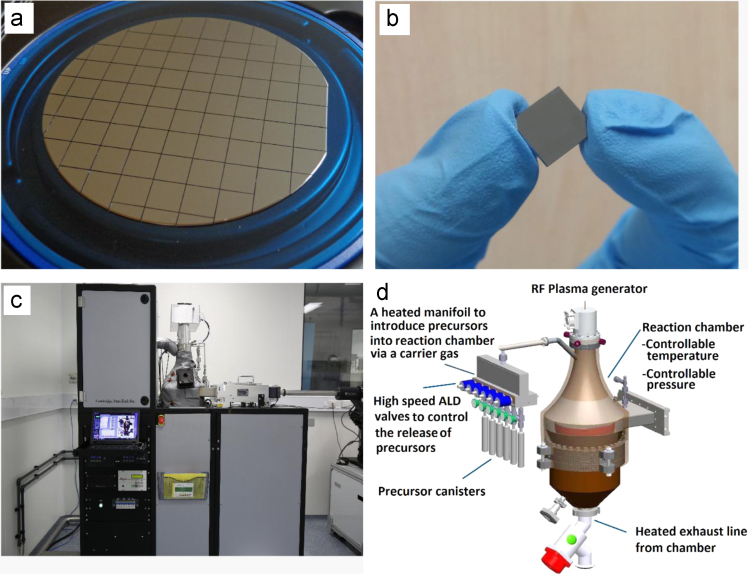
(a) Wafer-scale ALD TiO_2_ sample, (b) diced ~1.0×1.0 cm sample, (c) ALD apparatus and (d) graphical scheme of the ALD chamber.

Cambridge Nanotech ALD Fiji F200 was used as fabrication set-up. Plasma delivery was used to create the laminar flow of precursors. The wafer and the diced samples and the detailed parts of ALD instrument are show in Fig. 2.

### 2.3 Ellipsometric spectroscopy

Ellipsometry is an optical technique used mainly for investigating the dielectric properties and thicknesses of thin films. It is nondestructive, contactless, and used in many different fields, such as solid-state physics and microelectronics, for both fundamental research and industrial applications. Generally ellipsometry techniques act on the basis of theoretical and experimental aspects of anisotropy in sample which contains different layers. Simply changes in polarization of lights which are reflected form the surface are measured and expressed as two values (Ψ and Δ) which are attributed to ratio of Fresnel׳s reflection coefficients as defined in 1 [2]:

(1) $$\rho =\frac{r_{p}}{r_{s}}=\;\tan(\Psi )\;\exp\;(i\Delta )$$

Here r_s_ and r_p_ are respectively the complex Fresnel׳s reflection coefficients of the light polarized parallel (r_p_) and perpendicular (r_s_) to plane of incidence. A layer Cauchy model analysis was performed on measured ellipsometric angles. An example model is developed with a silicon substrate, a native SiO_2_ and an ALD deposited TiO_2_ layer above of the other layers, by using *CompletEase* program. The unknown properties of samples (thickness of TiO_2_) is defined as model “fit” parameter. The optical constant of Silicon, native SiO_2_ and TiO_2_ comes from library values [2]. The spectral dependencies of Ψ and Δ were fitted in the model to extract film thickness using a least square regression analysis and a weighted root mean square error (MSE) function. MSE describes the difference between experimental data and model predicted data. The normal fit is based on Levenberg-Marquart regression algorithm to minimize the least square difference between experimental and model generated data or in another words to minimize MSE which is defined in 2 [2], [3], [4]:

(2) $$MSE=\frac{1}{2N-M}\sum_{i=1}^{N}\left[{\left(\frac{\Psi_{i}^{\mathrm{mod}}-\Psi_{i}^{\exp}}{\sigma_{\Psi ,i}^{\exp}}\right)}^{2}+{\left(\frac{\Delta_{i}^{\mathrm{mod}}-\Delta_{i}^{\exp}}{\sigma_{\Delta ,i}^{\exp}}\right)}^{2}\right]=\frac{1}{2N-M}X^{2}$$

**Fig. 3:**
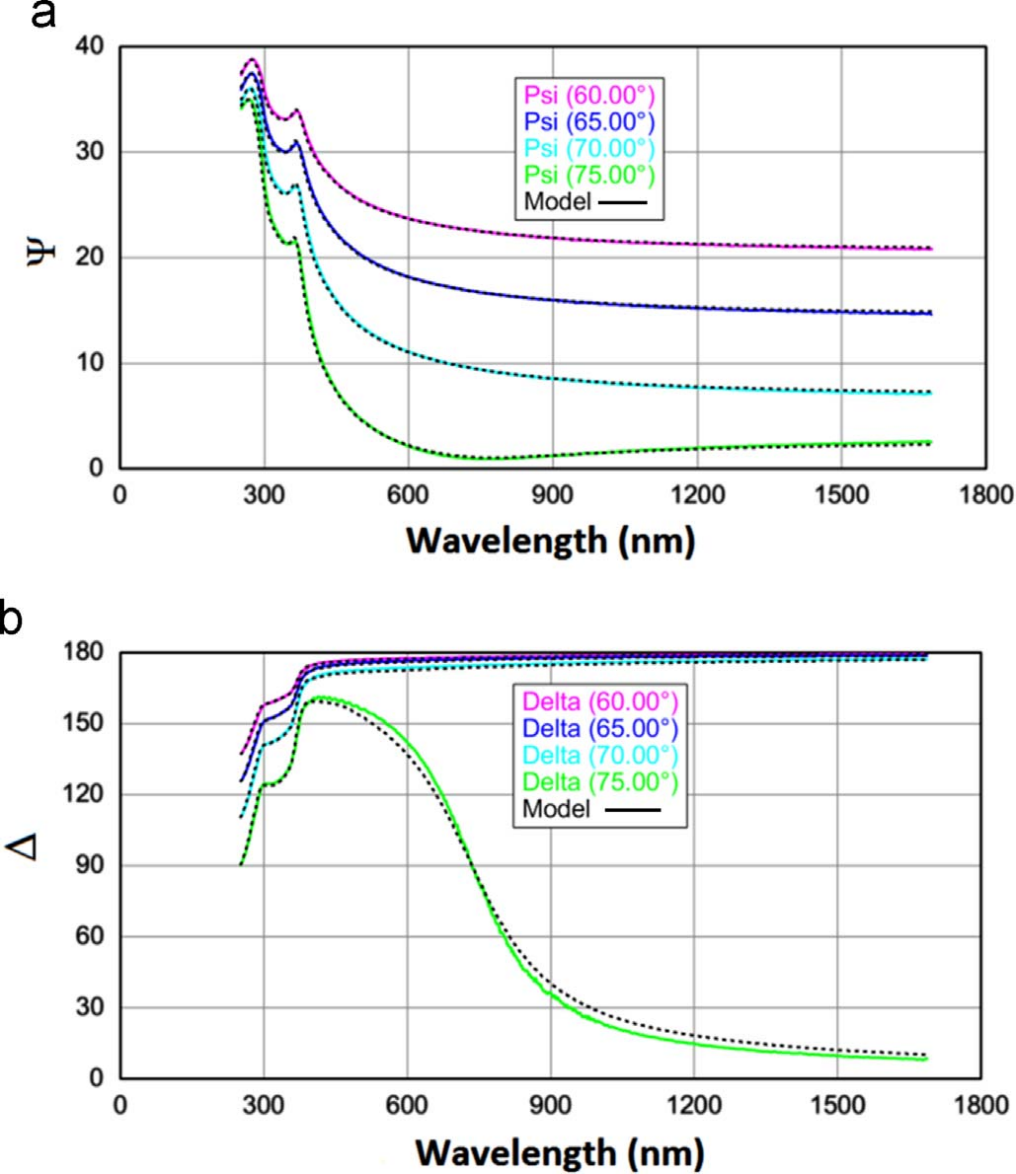
Spectral ellipsometric data (a) Ψ and (b) Δ vs. wavelength for atomic-layered TiO_2_ film.

Here “i” identifies each unique wavelength and angle of incidence, “N*”* is the total number of (Ψ,Δ) pairs, and “M” is the number of fit parameters. Furthermore, $\Psi_{i}^{\exp},\;\Delta_{i}^{\exp}$and $\Psi_{i}^{\mathrm{mod}},\;\Delta_{i}^{\mathrm{mod}}$ are experimental and modeled values of Ψ and Δ, respectively. Also, $\sigma_{\Psi ,i}^{\exp}$ and $\sigma_{\Delta ,i}^{\exp}$ are respectively the experimental standard deviation in Ψ and Δ . The spectral dependencies of Ψ and Δ for experimental and model generated data at various angle of incidence with corresponding dispersion relation are respectively presented in Fig. 3(a) and (b), where dashed lines represent practical data and continues lines show model generated data.

**Fig. 4:**
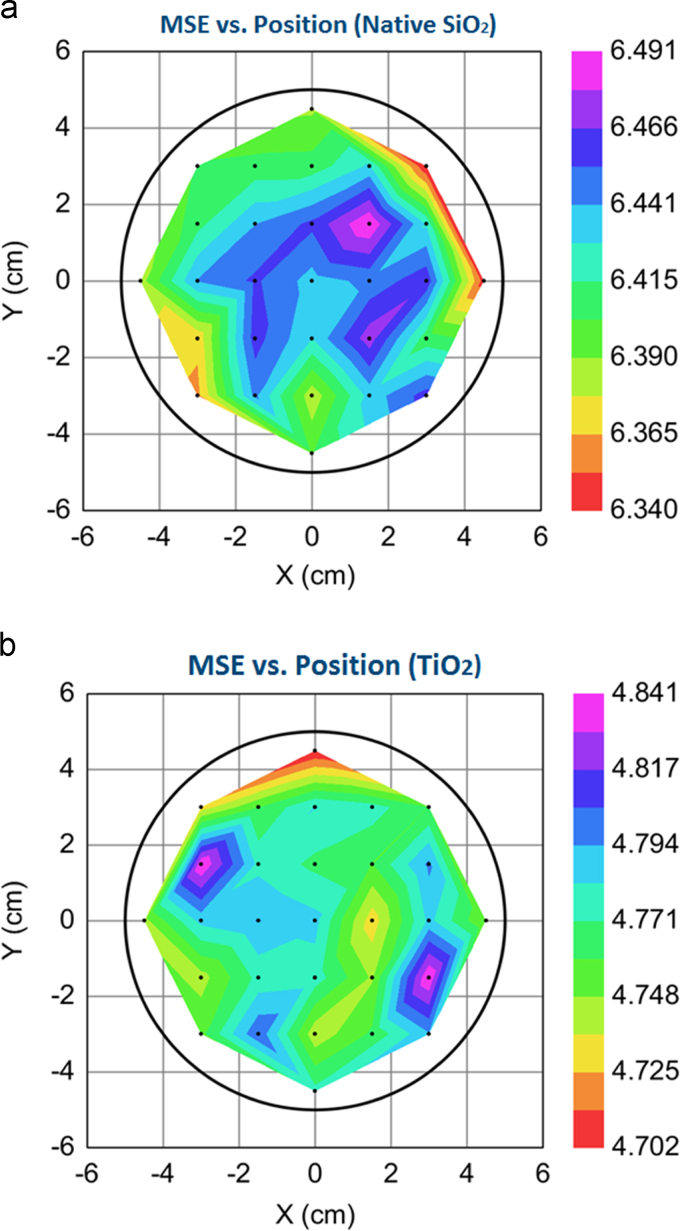
MSE for (a) native SiO_2_ and (b) atomic-layered TiO_2_ film.

Furthermore, fit results show that MSE is 6.3 arbitrary unite for native SiO_2_ and 4.78 arbitrary unite for as-deposited TiO_2_ layer indicating a perfect fit between practical and generated model data [3]. It should be mentioned that optical constants of TiO_2_ were unchanged from the library values meaning the only parameter that displays the difference between the deposited TiO_2_ and the library TiO_2_ is MSE value which is consistently less than 5 arbitrary unites [3]. Variations of MSE versus the position on the wafer surface for native SiO_2_ and as-deposited atomic-layered TiO_2_ are respectively shown in Fig. 4(a) and (b).

### 2.4 Atomic force microscopy measurements

**Fig. 5:**
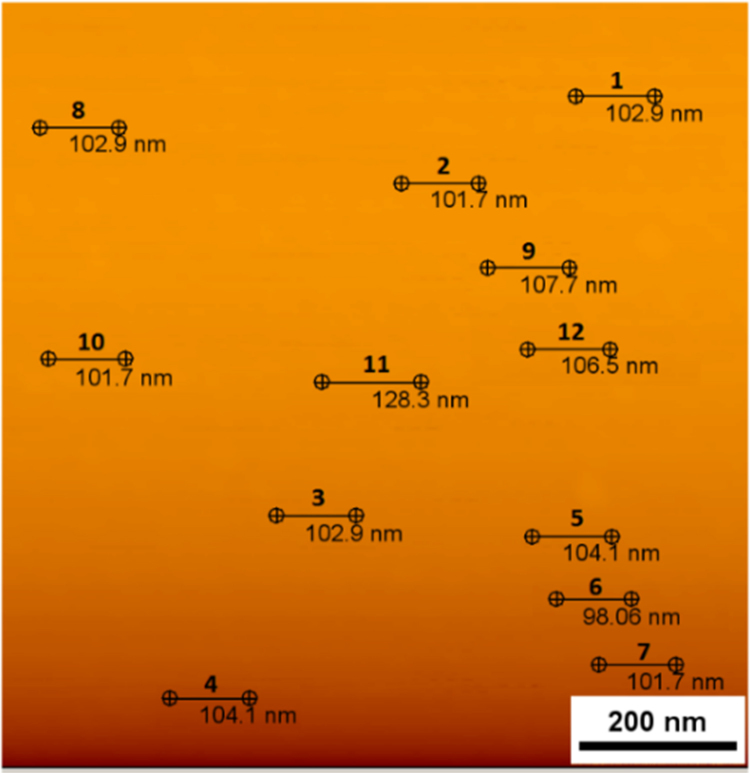
Height mode Image of AFM.

Fig. 5 shows the height mode image of AFM which is provided by the JPK data processing software (NanoWizard). To measure the average surface roughness and root mean square roughness values of surface, 12 lines with the length of 100 nm are measured. The data of roughness measurements is provided in Table 1, showing each specific line with its measured roughness.

**Table 1:** Data of root mean square roughness and average roughness of the atomic-layered film.

**Point**	**Root mean square roughness (pm)**	**Average roughness (pm)**
**1**	94.44	81.43
**2**	105.8	87.18
**3**	179.6	152.0
**4**	142.3	119
**5**	71.95	56.49
**6**	226.9	189.7
**7**	127.3	106.9
**8**	113.4	86.28
**9**	131.8	111.5
**10**	133.4	113.7
**11**	198.1	156.1
**12**	166.7	131.6
**Average**	**124.6**	**107.6**

### 2.5 Bandgap calculation

To estimate the optical bandgap of atomic-layered TiO_2_ film a general formula which relates the absorption coefficient (α) to the bandgap energy was used [5], [6]:

(3) $${\left(\alpha h\nu \right)}^{1/m}{=B(h\nu -\;\text{E}}_{g})$$

Where α is the absorption value in UV–vis spectrum, *h* is the Planck׳s constant, hν is the photon energy, m is the integer or semi integer, and E_g_ is the energy of the bandgap. B is a constant that depends on the transition probability. For m=2 the indirect transitions of electrons from the valence to the conduction bond are allowed [7], [8], [9]. Assuming that the transition probability is 1 (B=1), and $h\nu =h\frac{C}{\lambda }=\frac{1240}{\lambda }$, the Eq. (3) for indirect semiconductors could be simplified as presented below:

(4) $${\left(\alpha \frac{1240}{\lambda }\right)}^{1/2}=\frac{1240}{\lambda }-\;E_{g}$$

By treating ${\left(\alpha h\nu \right)}^{1/2}$ as Y axis and hν as the X axis, E_g_ can be estimated by extrapolating a straight line to the ${\left(\alpha h\nu \right)}^{1/2}=0$ axis in the plot of ${\left(\alpha h\nu \right)}^{1/2}$versus optical bandgap energy. The estimated bandgap is 3.37 eV.
