# Supplementary material for: Data set for fabrication of conformal two-dimensional TiO2 by atomic layer deposition using tetrakis (dimethylamino) titanium (TDMAT) and H2O precursors
Source: Data Brief. 2017 Jun 9;13:401–7. doi: 10.1016/j.dib.2017.06.013 (PMC5480828; doi:10.1016/j.dib.2017.06.013)
Supplement: Supplementary file 1 — Supplementary material [file mmc1.docx]

All authors declare no competing financial interests and conflict of interests.
